# Age at diagnosis predicted survival outcome of female patients with breast cancer at a tertiary hospital in Yogyakarta, Indonesia

**DOI:** 10.11604/pamj.2018.31.163.17284

**Published:** 2018-11-07

**Authors:** Evi Susanti Sinaga, Riris Andono Ahmad, Siddharudha Shivalli, Susanna Hilda Hutajulu

**Affiliations:** 1 Department of Community Medicine/Public Health, Faculty of Medicine, Trisakti University, Jakarta, Indonesia; 2 Department of Biostatistics, Epidemiology and Population Health, Faculty of Medicine, Public Health and Nursing, Universitas Gadjah Mada, Yogyakarta, Indonesia; 3 Department of Public Heath, Yenepoya Medical College, Yenepoya University, Mangalore, Karnataka, India; 4 Non-Communicable Diseases Regional Technical Advisor, Southeast Asia Regional Office (SEARO), TEPHINET, A Program of The Task Force for Global Health, Inc., Decatur, GA, United States of America; 5 Division of Hematology and Medical Oncology, Department of Internal Medicine, Faculty of Medicine, Public Health and Nursing, Universitas Gadjah Mada/Dr Sardjito General Hospital, Yogyakarta, Indonesia

**Keywords:** Breast cancer, five-year overall survival, age, Indonesia

## Abstract

**Introduction:**

Breast cancer is the most common cancer in women in Indonesia. Patients' survival depends on various factors, namely patient-, tumor-, and treatment-related factors. Survival analysis on Indonesian patients has only been reported in a few studies. This study aimed to identify the factors that are associated with five-year overall survival (OS) among patients with breast cancer at a local tertiary hospital in Indonesia.

**Methods:**

A retrospective cohort study was conducted at Dr Sardjito Hospital, Yogyakarta. Female patients diagnosed with breast cancer between January and December 2009 were studied. Socio-demographic and clinicopathological data were collected from the medical and pathological records. The five-year OS rate was assessed using Kaplan Meier method and prognostic factors were analyzed using Cox regression.

**Results:**

A total of 213 eligible patients with breast cancer were recruited. The five-year OS probability of the breast cancer patient was 51.07%. The majority of the patients (151, 70.9%) presented an advanced stage at the time of diagnosis. In the bi-variable analysis, cases who were younger, of a lower educational status, at a more advanced stage, with a bigger tumor size, and a central tumor location showed a worse five-year OS compared to their counterparts (p = 0.005, 0.001, 0.004, 0.011 and 0.023, respectively). In the multivariable analysis, age was an independent predictor for the OS (HR = 3.73; 95% CI = 1.0-13.6, p = 0.046).

**Conclusion:**

The five-year OS of breast cancer patients in the local tertiary hospital was 51.07%. The patients' age at diagnosis was the only significant prognostic factor for the patients' survival.

## Introduction

Breast cancer is the most common cancer diagnosed among women worldwide. Since 2008, the incidence of breast cancer has increased by > 20%, with an estimated 1.67 million of new breast cancer patients diagnosed in 2012. The majority of these cases occur in populations living in less developed regions. This malignancy ranks as the fifth most frequent cause of mortality among all types of cancers, with 522,000 deaths. It is the most frequent cause of cancer death in women from less developed countries (324,000 deaths) and the second most frequent cause of cancer death in more developed countries (198,000 deaths), after lung cancer [1, 2]. Breast cancer is a major public health problem in developing countries with limited resources, like Indonesia [3, 4]. Based on GLOBOCAN data, breast cancer in Indonesia is the most common cancer in women with an age-standardized incidence of 40.3/100,000 [1]. Data from the Jakarta Population-Based Cancer Registry (PBCR), that acted as a source for GLOBOCAN's data, showed that breast cancer was the top leading malignancy among Indonesian females from 2005 to 2007, followed by cervical cancer, ovarian cancer, colorectal cancer, bronchus and lung cancer, thyroid cancer, corpus uteri cancer, pharyngeal cancer, leukemia, and liver cancer [4]. According to the Health Ministry of Indonesia, the high incidence of advanced disease and mortality in breast cancer is due to the lack of a screening program, and poor access to, or the availability of, treatment. Most breast cancer patients seek healthcare only when they have symptoms and are diagnosed as being at an advanced stage [5]. Indeed, only 5% of women in developing countries like Indonesia were screened compared to 40% in advanced countries [6]. The survival of patients with breast cancer depends on many factors. Demographic variables such as age [7-10], educational level [11], financial status [12], family history [13] and marital status [14, 15] have been found to influence the survival rate. Tumor and clinicopathological parameters that have been observed to impact on survival include tumor size, nodal status, the presence of metastatic disease, the clincal stage, tumor location, histology grade, and the presence of comorbidities [9, 16-19]. Treatment factors that relate to the survival rate include the completeness of the approaches to treatment and surgical intervention [20, 21]. In fact, there are only a few reports of factors impacting on the survival of Indonesian women with breast cancer [8, 9, 22, 23]. Thus, the aim of the study was to identify the factors associated with the five-year overall survival (OS) among breast cancer patients at a local hospital. Analyzing the key factors would be of great use in identifying individuals at high risk of mortality and planning appropriate interventions.

## Methods

A retrospective cohort study was conducted at Dr Sardjito Hospital, which functions as a tertiary referral hospital in Yogyakarta, Indonesia. We included female breast cancer patients who were diagnosed in 2009. The patients were treated according to the standard protocol and prospectively followed to assess their survival over a period of five years. Details of the patients' information were extracted from the medical and pathological records, either from the medical records central unit, the cancer clinic, or the pathology laboratory. The following relevant data were collected: age at diagnosis, socio-demographic details (marital and educational status), family history of breast cancer, pathological diagnosis, clinical stage, tumor size, histology type, tumor location, comorbidity, and treatment. The survival status of the patients was obtained from the medical records at the end of five years of diagnosis. Telephone interviews were conducted for those who did not have life status in the medical records. A core family member of the patient was considered as a respondent if the patient had passed away. A semi-structured questionnaire was used. Data was considered as missing if the authors could not collect the life status data from medical records or had no response from three attempts to contact the patient. The patient characteristics were described as percentages. Kaplan-Meier constructs were conducted to estimate OS rates. The survival time of a patient referred to the number of months from the date of diagnosis to the date of the patient died, date of loss to follow-up, or date of the end of the study for patients who were still alive. All data were analyzed using STATA. Prior to analysis, data was edited, coded, entered, and cleaned in Microsoft excel [24]. Survival analysis was conducted by checking the assumption of proportional hazards (PH), bi-variable and multivariable analysis. PH assumption was determined by Kaplan-Meier curves [25]. For those variables which fulfilled the PH assumption, bi-variable cox regression analysis was performed [26, 27]. All variables with p <0.25 on bi-variable analysis were then entered into multivariable analysis. Four models were constructed based on the key variables and a model was selected by looking at p value and smallest Bayesian information criteria. This study was approved by the Ethics Committee of Medical and Health Research, Faculty of Medicine, Public Health and Nursing, Universitas Gadjah Mada/Dr Sardjito Hospital, Yogyakarta, Indonesia (reference number: KE/FK/690/EC/2015). Written informed and oral consent with digital recording were obtained during personal and telephone interviews to study participant or patient's relative.

## Results

**Figure 1:**
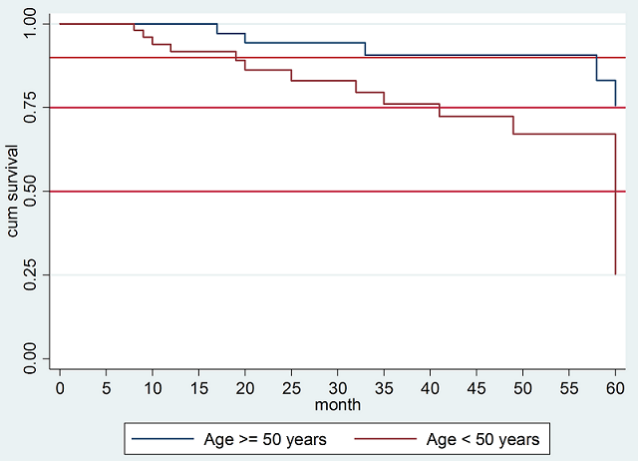
Kaplan-Meier plot comparing five-year overall survival (OS) between patients with breast cancer aged ≥50 years and <50 years (HR = 3.73; 95% CI = 1.0-13.6, p = 0.046)

A total number of 213 eligible female patients with breast cancer were recruited. Thirty patients (14.1%) were known to be alive five years after their diagnosis. The survival status of the remaining 183 patients (85.9%) is unknown or lost during the follow-up. Table 1 displays the key socio-demographic and clinicopathologic characteristics of all the patients. Nearly half of them (105, 49.3%) were aged < 50 years. As many as 86 (40.4%) and 44 patients (20.7%) were married and employed. One fifth of them (43, 20.2%) had completed a high school education or above. A family history of breast cancer was reported among 25 (11.7%) patients. Most of all cases (151, 70.9%) were presented in the advanced stage of disease at the time of diagnosis, with tumor size of ≥5cm (90, 42.3%). Invasive ductal carcinoma was the most frequent histological diagnosis (182, 85.4%) and the cancer location was central in 61 patients (28.7%). As many as 66 patients (31.0%) had comorbidities and more than half of the cases (125, 58.7%) underwent surgical intervention. Eighty percent of the patients (183) were censored due to failing to follow-up. Death was reported for 21 patients (9.9%) and 9 patients (4.2%) were reported as still being alive. The survival probability of breast cancer patients at the end of the five-year follow-up was 51.07%. In the bi-variable analysis, patients aged < 50 years, with a below high school education, advanced stage cancer, bigger tumor size, and central tumor location demonstrated a statistically significant worse five-year OS than their counterparts (p = 0.005, 0.001, 0.004, 0.011, and 0.023, respectively) (Table 2). In the multivariable analysis, four main models were constructed with a combination of three variables (age at diagnosis, clinical stage, and tumor size). From the four models, the combinations of age at diagnosis and tumor size were selected (Table 3). Finally, it was found that younger cases had a significantly increased probability of mortality compared to the older patients (Figure 1) (HR = 3.73; 95% confidence interval/CI = 1.0-13.6, p = 0.046).

**Table 1:** Characteristics of patients with breast cancer

Variables	Frequency (%)
**Age**	
≥50 years	104 (48.8)
<50 years	105 (49.3)
Missing data	4 (1.9)
**Marital Status**	
Married	86 (40.4)
Unmarried	5 (2.3)
Missing data	122 (57.3)
**Education**	
High school and above	43 (20.2)
Below high school	25 (11.7)
Missing data	145 (68.1)
**Family history of breast cancer**	
Yes	25 (11.7)
No	25 (11.7)
Missing data	163 (76.6)
**Stage**	
Advanced stage	151 (70.9)
Early stage	38 (17.8)
Missing data	24 (11.3)
**Tumor Size**	
≥5 cm	90 (42.3)
<5 cm	50 (23.5)
Missing data	73 (34.2)
**Histology type**	
Invasive ductal carcinoma	182 (85.4)
Invasive lobular and other carcinoma	13 (6.1)
Missing data	18 (8.5)
**Tumor location**	
Central	61 (28.7)
Periphery	42 (19.7)
Missing data	110 (51.6)
**Comorbidity**	
Yes	66 (31.0)
No	10 (4.7)
Missing data	137 (64.3)
**Surgical intervention**	
Yes	125 (58.7)
No	67 (31.5)
Missing data	21 (9.8)

**Table 2:** Bi-variable analysis for survival of patients with breast cancer

Variables	Frequency (%)	HR (95% CI)	P value
**Age**			
≥50 years	104 (48.8)	1.0 (ref)	0.005
<50 years	105 (49.3)	3.6 (1.3-9.10)	
Missing data	4 (1.9)		
**Marital Status**			
Married	86 (40.4)	1.0 (ref)	0.105
Unmarried	5 (2.3)	5.04 (0.6-44.5)	
Missing data	122 (57.3)		
**Education**			
High school and above	43 (20.2)	1.0 (ref)	0.001
Below high school	25 (11.7)	5.31 (1.8-15.8)	
Missing data	145 (68.1)		
**Family history of breast cancer**			
Yes	25 (11.7)	1.0 (ref)	0.690
No	25 (11.7)	1.25 (0.3-4.7)	
Missing data	163 (76.6)		
**Stage**			
Advanced stage	151 (70.9)	1.0 (ref)	0.004
Early stage	38 (17.8)	0.16 (0.04-0.7)	
Missing data	24 (11.3)		
**Tumor size**			
≥5 cm	90 (42.3)	1.0 (ref)	0.011
<5 cm	50 (23.5)	0.19 (0.04-0.9)	
Missing data	73 (34.2)		
**Histology type**			
Invasive ductal carcinoma	182 (85.4)	1.0 (ref)	0.614
Invasive lobular and other carcinoma	13 (6.1)	1.47 (0.3-6.7)	
Missing data	18 (8.5)		
**Tumor location**			
Central	61 (28.7)	1.0 (ref)	0.023
Periphery	42 (19.7)	0.23 (0.1-0.9)	
Missing data	110 (51.6)		
**Comorbidity**			
Yes	66 (31.0)	1.0 (ref)	0.296
No	10 (4.7)	2.34 (0.5-12.2)	
Missing data	137 (64.3)		
**Surgical intervention**			
Yes	125 (58.7)	1.0 (ref)	0.519
No	67 (31.5)	1.47 (0.4-5.1)	
Missing data	21 (9.8)		

HR: hazard ratio; CI: confidence interval

**Table 3:** Multivariable analysis of various factors associated with survival of patients with breast cancer

Variable	Model 1		Model 2		Model 3		Model 4*	
	HR (95% CI)	P value	HR (95% CI)	P value	HR (95% CI)	P value	HR (95% CI)	P value
Stage	0.29 (0.0-2.7)	0.279	0.24 (0.1-1.1)	0.065	0.16 (0.0-1.3)	0.089	-	
Age at diagnosis	2.66 (0.7-10.4)	0.160	2.71 (0.9-7.6)	0.059	-		3.73 (1.0-13.6)	0.046
Tumor size	0.36 (0.1-1.7)	0.204	-		0.35 (0.1-1.7)	0.194	0.29 (0.1-1.4)	0.124
BIC	106.27		152.55		103.85		104.54	

HR: hazad ratio; CI: confidence interval; BIC: Bayesian information criteria

final model

## Discussion

Survival of breast cancer in Indonesian patients is not very frequently analyzed. Indeed, some previous reports have come from our local institution. Aryandono *et al.* (2006) determined prognostic factors in operable patients and selected cases [8, 9]. Another publication by Widodo *et al.* (2017) recently showed prognostic determinants for patients diagnosed in 2008-2013 with different molecular types [22]. Also recently, a study from another institution identified a relationship between disease free survival and OS rates with the duration of targeted therapy in HER-2 positive operable patients [23]. However, those studies recruited only selected patients in different cohorts. Our present study analyzed a whole cohort of subjects, diagnosed in 2009, to see the patients' five-year OS regardless of their clinical stage and pathological characters. Exclusion was only done for subjects with very minimal data. Our findings showed that the five-year OS rate of local breast cancer patients was 51.07%. This was similar to those reported in India (52%), Brazil (58%), Uganda (46%), and Malaysia (49%). Nevertheless, this rate was much lower than those reported in developed countries, such as China (82%), Korea (79%), Cuba (84%), and Costa Rica (70%) [28-30]. Estimated survival rates vary according to the follow-up time period, the study population, the quality of the data, the statistical method used, and the possibility of biases. They are also affected by screening programs and the availability and accessibility of timely treatment. These suggest that the comparison of survival rates should be interpreted with caution [31]. Despite having data collected from many resources, the present study had a lot of missing information. As a consequence, many factors that might affect the survival rate, such as the precise follow-up period and the completeness of the treatment could not be accurately determined. However, the data still laid a base for the study's conclusion and provided a general picture of the local situation. Just recently, Yogyakarta cancer registry has been developed to be part of the nationwide population-based cancer registry. In the future, data recording and data collection should be improved, which will provide better information for upcoming studies and advocation to the authorities for cancer management. One other reason that may influence the low survival rate in local patients is the fact that most of the cases were presented in an advanced stage at the baseline. This strongly emphasizes the need for a screening program among the local women. As suggested by Shulman *et al.* early detection by screening, which is subsequently followed by appropriate treatment, is needed to increase the survival rate of patients [32].

In the bi-variable analysis, the present study demonstrated that patients with a lower education level, a more advanced stage of cancer, a bigger tumor, and a central tumor location were at a higher risk of mortality. The level of education has an influence on the knowledge and behavior of the patients. Patients with a good education tend to have a better comprehension of a variety of information and hence better knowledge and favorable behavior [11]. Similarly, the clinical stage of breast cancer and tumor size at the time of diagnosis are indeed important prognostic factors affecting survival [8, 9, 17, 18]. Tumor volume indicates the spread of the disease into surrounding areas and larger tumors show greater chances of spreading to other areas [33]. This is clinically often associated with a palpable regional lymph node. In addition, large tumors are frequently linked with a higher risk of relapse, irrespective of the lymph node status. Likewise, centrally located tumors have the tendency to spread to internal organs through local lymph nodes and correlate with a poor prognosis [16]. Many cohorts have observed that younger age is an independent predictor of an unfavorable treatment response, more aggressive tumor behavior, and a decreased survival rate [7-10, 34]. Younger women with breast cancer have a high tumor grade, clinically are more aggressive, present in an advanced stage, and have a high risk of recurrence [7, 10, 35]. Tumors in younger women tend to be negative for hormone receptors, and thus show a poor response to adjuvant therapy [7]. Supporting those findings, our study found that being < 50 years old at the baseline was associated with an increased risk of mortality. Eventually, age was the only independent prognostic variable for the patient's five-year OS in the multivariable analysis. In contrast, other studies found that elderly women with breast cancer also have poor outcomes [16, 19, 36]. Differences in the subjects' characteristics across multiple studies may provide some reasons for these disagreements.

## Conclusion

The five-year OS of breast cancer patients in the local tertiary hospital was 51.07%, which is relatively low compared to the survival of patients in developed countries. Screening implementation, an improved diagnostic approach, and increased access to treatment must be made available to enhance the survival of our local patients. Among many prognostic factors, age at diagnosis was the only significant predictor for the survival outcome, indicating that younger cases may need more attention and more aggressive treatment compared to older people. This also demands further studies to explore the risk factors in the local population, in particular in relation to their age.

### What is known about this topic

- Breast cancer is the most prevalent cancer in Indonesia;
- Some studies showed the survival of Indonesian patients with breast cancer in selected population.

### What this study adds

- This study showed survival of all patients of a tertiary hospital in Indonesia diagnosed in 2009 (without selection);
- Age, educational status, stage, tumor size and tumor location are factors affecting the five-year overall survival;
- Age is found as independent prognostic factor for the overall survival.

## Competing interests

The authors declare no competing interests.
